# Lymph node metastasis of papillary thyroid carcinoma in the context of Hashimoto’s thyroiditis

**DOI:** 10.1186/s12902-021-00923-2

**Published:** 2022-01-05

**Authors:** Lirong Wang, Jiawen Chen, Xin Yuan, Juan Wang, Lei Sun, Jue Jiang, Lin Zhang, Min Liu, Qi Zhou

**Affiliations:** 1 Department of Ultrasound, the Second Affiliated Hospital of Xi’an Jiaotong University, Xi’an, 710004 China; 2 Department of Otolaryngology Head and Neck Surgery, the Second Affiliated Hospital of Xi’an Jiaotong University, Xi’an, 710004 China

**Keywords:** Hashimoto’s thyroiditis, Papillary thyroid carcinoma, Lymph node metastasis, Predictive model

## Abstract

**Background:**

Whether Hashimoto’s thyroiditis (HT) affects the lymph node metastasis of papillary thyroid carcinoma (PTC) remains uncertain. The diagnostic criteria for HT differed in previous studies. Our study focused on analysing the influence of HT on PTC lymph node metastasis (LNM) with stringent diagnostic criteria for HT.

**Methods:**

A total of 444 patients diagnosed with PTC from 2019 to 2020 were enrolled and divided into two groups: HT group and non-HT group. Diagnostic criteria of HT were as follows: thyroid peroxidase antibody (+) and postoperative histopathology of Hashimoto’s disease.

**Results:**

There was no significant difference in the LNM rate between HT group and non-HT group. Patients in the HT group had fewer numbers of metastatic LNs and lower metastatic LNs ratio in central region. In the HT group, age < 55 and tumor size ≥10 mm were independent risk factors for central LNM.

**Conclusion:**

The autoimmune response of HT seems to reduce the central lymph node metastasis of HT PTCs. Age < 55 and tumor size ≥10 mm were independent risk factors of central lymph node metastasis in HT PTCs.

**Supplementary Information:**

The online version contains supplementary material available at 10.1186/s12902-021-00923-2.

## Introduction

The incidence of thyroid cancer has continued to rise for decades. As of 2018, thyroid cancer ranked ninth among all cancers in the world [1]. The most common pathologic subtype is papillary thyroid carcinoma (PTC) [2], which is always associated with favorable overall survival rate. However, Lim et al. [3] have found that the incidence-based mortality of PTC actually increased. The causes for rising mortality are unknown, but may be similar to the causes of the rising incidence— environmental factors [4, 5]. In addition, the recurrence rate of PTC ranges from 20 to 40% [6], affecting patients’ disease-free survival. Lymph node metastasis (LNM) is associated with recurrence [7], and approximately 14–64% of PTC patients have regional LNM [8]. Therefore, analysing potential risk factors for LNM is beneficial to manage the risk stratification of PTC recurrence.

Hashimoto’s thyroiditis (HT) is a major thyroid autoimmune disease [9]. The human immune response of HT is destructive, eventually leading to thyroid failure [10], though the microenvironment surrounding PTC is immunosuppressed and promotes tumor growth [11]. Whether HT may affect the aggressiveness of PTC when the two diseases coexist is an intriguing topic. Some consider positive thyroid autoantibodies to be risk factors for cervical LNM in PTC [12, 13]. Others believe that HT PTCs have a low LNM rate and a better prognosis [14, 15]. However, some studies have suggested that HT has no protective effect on PTC [16]. Overall, the association between HT and LNM in PTC remains controversial. In addition, some PTC patients are found to have lymphocytic infiltration on postoperatively histopathologic examination, even though thyroid peroxidase antibody (TPO-Ab) or ultrasound signs are negative. The diagnosis of HT in these patients may not be prudent.

Through strict diagnostic criteria of HT—preoperative positive serum TPO-Ab and postoperative histopathology of Hashimoto’s disease—we conducted detailed stratified analysis and established predictive models to reveal the connection between HT and LNM in PTC.

## Methods

### Patients

With the approval of the Institutional Review Board of Xi’an Jiaotong University Second Affiliated Hospital, clinical data for PTC patients who underwent thyroid surgery at Second Affiliated Hospital of Xi’an Jiaotong University from January 2019 to December 2020 were screened. Patients with a postoperative pathological diagnosis of PTC were divided into two groups: those with Hashimoto’s thyroiditis (HT group) and those without Hashimoto’s thyroiditis (non-HT group). All patients underwent prophylactic central lymph node (LN) dissection. Suspicious lateral cervical LNs were confirmed by ultrasound-guided fine-needle aspiration, and the corresponding LN area was dissected. The inclusion criteria for the HT group were as follows: (1) preoperative TPO-Ab (+) and (2) histopathological diagnosis of Hashimoto’s thyroiditis. The inclusion criteria for the non-HT group were as follows: (1) negative preoperative TPO-Ab and (2) postoperative histopathology without Hashimoto’s thyroiditis. The exclusion criteria were as follows: (1) Other types of thyroid tumors or recurrent PTC; (2) received radiotherapy or thyroid-related drug treatment before surgery; (3) Graves’ disease or positive preoperative thyrotropin receptor antibody; and (4) incomplete or unavailable clinical and pathological data .

Pathological results were diagnosed by the same group of pathologists. The following pathological data were recorded: tumor size (if tumor was multifocal, the maximum diameter of the largest nodule was recorded), multifocality (yes/no), central region LNM (positive/negative), lateral region LNM (positive/negative), extrathyroidal extension (yes/no), and distant metastasis (yes/no). The patients were divided into stage I, stage II and stage III according to TNM staging of differentiated and anaplastic thyroid carcinoma of American Joint Committee on Cancer [17].

### Statistical analysis

SPSS 20.0 software (Chicago, IL) was used for statistical analysis. The median (first quartile, third quartile) was used to describe data with skew distribution. Other descriptive data are presented as mean ± standard deviation (SD). Categorical data are given as frequencies and proportions. Independent sample T test, Mann-Whitney U test, χ2 test, and Fisher exact test were used to analyse the data. Binary logistic regression was applied to reveal factors associated with cervical LNM. A prediction model of logistic regression was established to predict lymph node metastasis. In univariate analysis, variables with *P* < 0.2 were included in multivariate analysis. *P* values were derived from two-tailed tests; *P* < 0.05 was considered statistically significant.

## Results

### Total patient characteristics

**Fig. 1:**
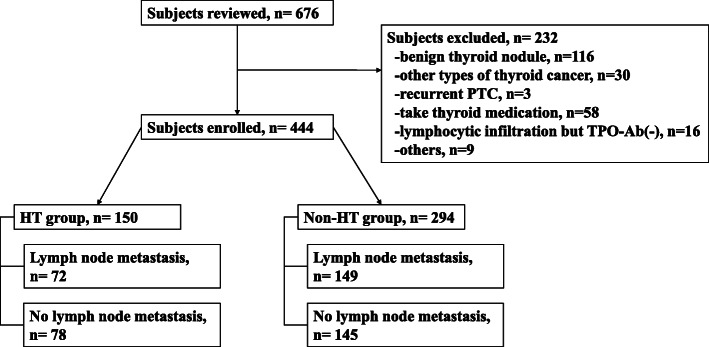
The selection and exclusion processes

The selection and exclusion processes are shown in Fig. 1. A total of 444 PTC patients were enrolled in the study, including 116 males and 328 females, with an average age of 44.2 ± 11.7 years. The median of tumor size was 9.8 mm. Among 444 cases, 133 patients (30.0%) had multifocal lesion on histopathology. A total of 221 patients had LNM; 170 (38.3%) had only central LNM, 7 (1.6%) had only lateral LNM, and 44 (9.9%) had both central and lateral LNM. Extrathyroidal extension was identified in 6 patients (1.4%). No patients developed distant metastasis. According to the AJCC eighth Edition - Staging Manual for Differentiated and anaplastic thyroid carcinoma, there were 401 (90.3%) stage I, 37 (8.3%) stage II, and 6 (1.4%) stage III patients (Table 1).

**Table 1:** Clinical and pathological characteristics of PTC patients

Characteristic	Total (*n* = 444)	HT (*n* = 150, 33.8%)	Non-HT (*n* = 294, 66.2%)	*P*
**Age, years**	44.2 ± 11.7	42.3 ± 11.4	45.1 ± 11.7	0.02^a^
**Sex, n (%)**				< 0.01^b^
Male	116 (26.1)	17 (11.3)	99 (33.7)	
Female	328 (73.9)	133 (88.7)	195 (66.3)	
**TSH level, μIU/mL**		3.49 (1.97,5.20)	2.23 (1.55,3.31)	< 0.01^c^
**TPO-Ab level, IU/mL**		184.15 (82.40,427.55)	11.00 (9.00,15.02)	< 0.01^c^
**TG-Ab level, IU/mL**		284.20 (73.99,554.65)	12.00 (10.00,18.20)	< 0.01^c^
**Tumor-size, mm**	9.80 (6.90,14.08)	9.00 (7.00,13.00)	10.00 (7.00,14.00)	0.04^c^
**Papillary microcarcinoma, n (%)**				0.04^b^
No	207 (46.6)	59 (39.3)	148 (50.3)	
Yes	237 (53.4)	91 (60.7)	146 (49.7)	
**Multifocality, n (%)**				0.83^b^
Yes	133 (30.0)	46 (30.7)	87 (29.6)	
No	311 (70.0)	104 (69.3)	207 (70.4)	
**Lymph node metastasis, n (%)**				0.93^d^
Positive	221 (49.8)	72 (48.0)	149 (50.7)	
Central only	170 (38.3)	57 (38.0)	113 (38.4)	
Lateral only	7 (1.6)	2 (1.3)	5 (1.7)	
Central+ Lateral	44 (9.9)	13 (8.7)	31 (10.5)	
Negative	223 (50.2)	78 (52.0)	145 (49.3)	
**Extrathyroidal extension, n (%)**				1.00^d^
Yes	6 (1.4)	2 (1.3)	4 (1.4)	
No	438 (98.6)	148 (98.7)	290 (98.6)	
**Distant metastasis, n (%)**	0 (0)			
**TNM Stage, n (%)**				< 0.01^d^
I	401 (90.3)	144 (96.0)	257 (87.4)	
II	37 (8.3)	4 (2.7)	33 (11.2)	
III	6 (1.4)	2 (1.3)	4 (1.4)	

^a^Student t test

^b^χ^2^ test

^c^Mann-Whitney U test

^d^Fisher’s exact test

### Comparison of clinical features between HT group and non-HT group

Most patients in the HT group were female (*p* < 0.05), were younger (*p* = 0.02) and had high levels of TSH, TPO-Ab and Tg-Ab (*p* < 0.05). In the HT group, tumor size was smaller (*p* = 0.04). Compared with the non-HT group, the majority of PTC patients in HT group were at TNM stage I (96% vs 87.4%, *p* < 0.05). Rates of LNM, multifocality and extrathyroidal extension between the two groups were not significantly different (Table 1).

### Association between HT and LNM

Table 2 shows that 214 patients had central LNM; 51 had lateral LNM. Patients in the HT group had more dissected LNs but fewer metastatic LNs in the central area (*p* < 0.05), with a lower metastatic LN ratio (*p* < 0.01). The number of LNs in the lateral region did not reach statistical difference. HT was not an independent risk factor for PTC LNM, neither central region nor lateral region (Supplemental Table 1, *p* > 0.05).

**Table 2:** Number of metastatic LNs in the two groups

	HT	Non-HT	*P*^a^
**Central LNM**			
Patients	70	144	
Number of dissected LNs	8.8 ± 4.9	7.2 ± 4.3	0.02
Number of metastasis LNs	3.0 ± 2.4	4.3 ± 4.3	0.01
Ratio, metastasis/ dissected LNs	0.38 ± 0.27	0.54 ± 0.33	< 0.01
**Lateral LNM**			
Patients	15	36	
Number of dissected LNs	12.8 ± 6.9	13.9 ± 8.1	0.82
Number of metastasis LNs	4.1 ± 2.5	4.3 ± 3.9	0.63
Ratio, metastasis/ dissected LNs	0.32 ± 0.13	0.32 ± 0.17	0.97

^a^Student t test

Table 3 shows independent risk factors for central LNM in the HT group were age < 55 and tumor size ≥10 mm, in the non-HT group were male sex and tumor size ≥10 mm. In the HT group, age correlated negatively with central LNM (OR 0.17, 95%CI: 0.05–0.55, *p* < 0.01), tumor size correlated positively with central LNM (OR 2.69, 95%CI: 1.32–5.49, *p* = 0.01).

**Table 3:** Predictive factors for central LNM in the HT and the non-HT group

Variables of central lymph node metastasis	HT						Non-HT					
	Univariate analysis			Multivariate analysis			Univariate analysis			Multivariate analysis		
	Crude OR	95%CI	*P*	Adjusted OR	95%CI	*P*	Crude OR	95%CI	*P*	Adjusted OR	95%CI	*P*
**Age, year** (< 55 / ≥55)	0.20	0.06–0.60	0.01	0.17	0.05–0.55	< 0.01	0.79	0.46–1.34	0.38			
**Sex** (Male, Female)	0.75	0.27–2.07	0.58				0.41	0.25–0.67	< 0.01	0.36	0.21–0.61	< 0.01
**TSH, μIU/mL** HT: 3.49< / ≥3.49 Non-HT: 2.23< / ≥2.23	0.81	0.42–1.53	0.51				1.39	0.88–2.20	0.16	1.53	0.94–2.51	0.09
**Tumor size, mm** (≤10 / > 10)	2.62	1.34–5.15	0.01	2.69	1.32–5.49	0.01	2.80	1.75–4.49	< 0.01	2.98	1.82–4.88	< 0.01
**Multifocality** (No / Yes)	2.02	1.00–4.08	0.05	1.96	0.92–4.17	0.08	1.42	0.86–2.35	0.17	1.23	0.72–2.11	0.45
**Extrathyroidal extension** (No / Yes)	/	/	1.00				0.34	0.04–3.33	0.36			

*OR* odds ratio, *CI* confidence interval

### Prediction model of central LNM for the HT group

**Fig. 2:**
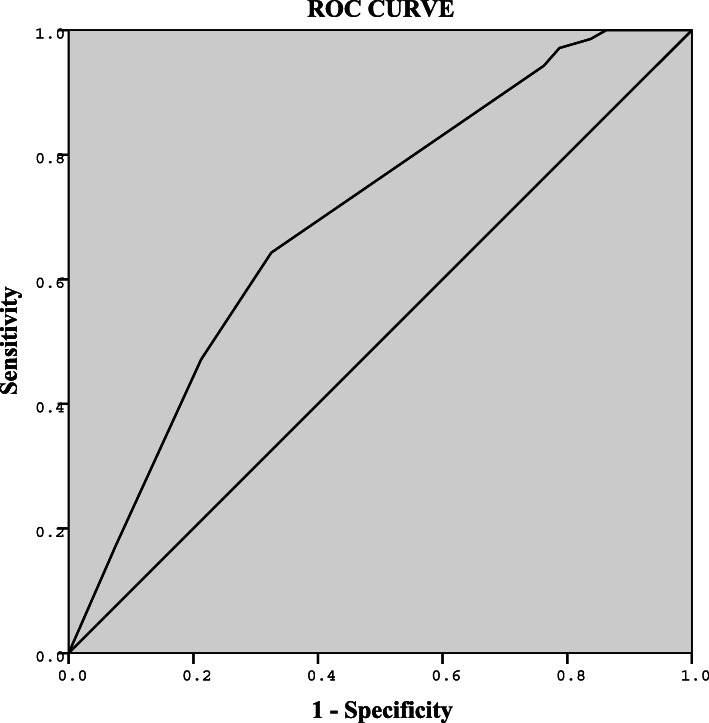
ROC curve of central LNM prediction model in the HT group. Logit (π) = −0.50-1.78*X1 + 0.99*X2, AUC = 0.70 (95%CI, 0.61–0.78). Cut-off value = 0.46, Youden index = 0.32, Sensitivity = 64%, Specificity = 68%, PPV = 63%, NPV = 68%. X1, age; X2, tumor size. π, the conditional probability of a positive result (central lymph node metastasis). AUC, area under the curve

Table 4 shows independent risk factors for central LNM in the HT group. In multivariate logistic analysis, age < 55 and tumor size ≥10 mm were independent risk factors for central LNM. There were no correlations between sex, TSH level, TPO-Ab level, Tg-Ab level, multifocality, extrathyroidal extension and central LNM. Figure 2 illustrates ROC curve of logistic regression model for predicting central LNM (AUC = 0.70, 95%CI: 0.61–0.78). The cut-off value was defined as 0.46 (sensitivity = 64%, specificity = 68%, PPV = 63%, NPV = 68%).

**Table 4:** Predictive factors of central LNM in the HT group

Variables	Central lymph node metastasis					
	Univariate analysis			Multivariate analysis		
	OR	95%CI	*P*	OR	95%CI	*P*
**Age, year** (< 55 / ≥55)	0.20	0.06–0.60	< 0.01	0.17	0.05–0.55	< 0.01
**Sex** (Male, Female)	0.75	0.27–2.07	0.58			
**TSH, median** (3.49< / ≥3.49, μIU/mL)	0.81	0.42–1.53	0.51			
**TPO-Ab, median** (184.15< / ≥184.15, IU/mL)	0.81	0.42–1.53	0.51			
**Tg-Ab, median** (284.2< / ≥284.2, IU/mL)	1.11	0.59–2.12	0.74			
**Tumor size, mm** (≤10 / > 10)	2.62	1.34–5.15	0.01	2.69	1.32–5.49	0.01
**Multifocality** (No / Yes)	2.02	1.00–4.08	0.05	1.96	0.92–4.17	0.08
**Extrathyroidal extension** (No / Yes)	0.46	0.39–0.55	0.22			

*OR* odds ratio, *CI* confidence interval

## Discussion

In recent years, the incidence of HT and PTC has both displayed an upwards trend. There is a potential association between the two diseases, but whether HT affects invasiveness of PTC is controversial. Some believe that HT is a protective factor for thyroid cancer [15, 18], whereas others propose the opposite [12, 13]. In our study, 33.8% of PTC patients had HT, consistent with the range of 0.4–42.5% reported previously [19–22]. Most HT PTCs were associated with female sex, younger age, smaller tumor size, better TNM stage and elevated TSH, TPO-Ab, and Tg-Ab levels. This is consistent with other studies [18, 23].

The aggressive clinical behaviours of PTC manifest as LNM and extrathyroidal extension. In our study, rate of extrathyroidal extension between the groups was not significantly different. Nor was the extrathyroidal extension a risk factor for LNM, which was different from Mao et al. [8]. This may be due to the small number of such cases. In addition, we observed that rate of LNM between the HT and non-HT groups was not significantly different. However, HT affected the number and positive rate of metastatic LNs in the central region to a certain extent. After multivariate analysis, the independent risk factors for PTC central LNM were age < 55, male sex and tumor size ≥10 mm, which was consistent with Mao et al. [8]. However, central LNM was independent of HT state and TSH level. This conclusion was partly consistent with the research of Kim et al. [18], who suggested that HT is a protective factor for central LNM. This different results may be due to the different diagnostic criteria we adopted for HT. In the study of Kim et al., HT was diagnosed when one of the following criteria was met: (1) TPO-Ab (+), (2) Tg-Ab (+), or (3) pathological diagnosis of Hashimoto’s disease. We only enrolled in the HT group patients with both TPO-Ab positivity and pathological diagnosis of HT. In fact, TPO-Ab and Tg-Ab may be positive in 10–15% of people without autoimmune thyroid diseases [24]. Differentiated thyroid cancer may also cause an increase in Tg-Ab [25]. In general, different inclusion criteria may have an impact on the results.

Previous studies have reported that young age is a risk factor for PTC LNM, while female sex is a protective factor [26, 27]. In our study, most patients in the HT group were younger females; thus, risk and protective factors were mixed. The independent risk factors for central LNM in the HT group were age < 55 and tumor size ≥10 mm, in the non-HT group were male sex and tumor size ≥10 mm. Male sex is an independent risk factor for PTC central LNM in other studies [8, 26–28]. However, this association disappeared in HT PTCs. The damage of human immune response to thyroid cells in Hashimoto’s thyroiditis is destructive [10]. We hypothesize that diffuse infiltration of lymphocytes around the lesion may reduce central LNM to some extent in HT PTCs. This potential protective effect appeared to be more significant in male sex with HT PTCs; thus, male sex was no longer an independent risk factor of central LNM in HT PTCs. Clinicians should pay more attention to age and tumor size in HT PTCs when evaluating central lymph node metastasis. Our regression model shows an acceptable PPV of 66%, which still needs to incorporate other molecular markers to enhance prediction efficiency. In differentiated thyroid cancer, the most common genetic changes include BRAF and RAS mutations and RET rearrangement. Although BRAF^V600E^ mutation is clearly associated with clinical outcome, while its accuracy is limited by low specificity [29]. Marotta V et al. [30] have found that VEGF-A SNPs are related to the prognosis of differentiated thyroid cancer. The inclusion of characteristic molecular markers may improve accuracy and specificity of the prediction model, and this needs further verification.

Previous studies have found that low ratio of metastatic LNs predicted a low risk of recurrence and better disease-specific survival [31–33]. In our study, patients in the HT group had lower TNM stages and number and ratio of metastasis LNs. Based on previous analysis, we believe that the lower TNM stage is due to the younger age and smaller tumor size in these cases. In addition, a possible explanation for the smaller tumor size and fewer metastatic LNs is that lymphocytic infiltrates of HT prevented tumor cells from growing and metastasizing to lymph nodes to some extent, such as infiltrates of activated NK cells and macrophages (M1/killer phenotype) [34]. We speculate that HT PTCs may be more indolent, that these patients may have a lower recurrence rate and excessive treatment may be needless.

Our study was a cross-sectional study and therefore could not determine the causal relationship between each factor and LNM. As the patients we enrolled were not receiving thyroid-related medication, impairment of thyroid function in the HT group may be mild. This may bring a certain selection bias. We also analysed the influence of TSH, TPO-Ab and Tg-Ab on central LNM in the HT group, revealing no significant effect. The prediction model for central LNM in HT PTCs still need to be improved. We will continue to follow up with the patients in this study to further investigate the prognosis of HT PTCs. In addition, BRAF gene mutations are associated with LNM in PTC, and it is necessary to conduct research on these mutations in PTC patients with HT.

## Conclusions

In conclusion, HT does not significantly affect the rate of lymph node metastasis. However, the autoimmune response of HT seems to reduce the central lymph node metastasis in HT PTCs to some extent. When evaluating central LNM in HT PTCs, clinicians should focus on age and tumor size.

## Supplementary Information

**Additional file 1.**

## Data Availability

The clinical data of our study are available from the corresponding author on reasonable request.

## Abbreviations

HT: Hashimoto’s thyroiditis
PTC: Papillary thyroid carcinoma
TSH: Thyroid-stimulating hormone
TPO-Ab: Thyroid peroxidase antibody
Tg-Ab: Thyroglobulin antibody
LNM: Lymph node metastasis
LN: Lymph node
