# Supplementary material for: Lymph node metastasis of papillary thyroid carcinoma in the context of Hashimoto’s thyroiditis
Source: BMC Endocr Disord. 2022 Jan 5;22:12. doi: 10.1186/s12902-021-00923-2 (PMC8734374; doi:10.1186/s12902-021-00923-2)
Supplement: Supplementary file 1 — Additional file 1. [file 12902_2021_923_MOESM1_ESM.doc]

**Supplementary Table1.** Univariate and multivariate analysis of LNM in patients with PTC

| **Variables** | **Central lymph node metastasis** | | | | | | **Lateral lymph node metastasis** | | | | | |
| --- | --- | --- | --- | --- | --- | --- | --- | --- | --- | --- | --- | --- |
|  | **Univariate analysis** | | | **Multivariate analysis** | | | **Univariate analysis** | | | **Multivariate analysis** | | |
|  | **OR** | **95％CI** | **P** | **OR** | **95％CI** | **P** | **OR** | **95％CI** | **P** | **OR** | **95％CI** | **P** |
| **Age, year**  (<55 / ≥55) | 0.58 | 0.36-0.92 | 0.02 | 0.57 | 0.35-0.94 | 0.03 | 0.53 | 0.24-1.19 | 0.13 | 0.39 | 0.16-0.97 | 0.04 |
| **Sex**  (Male, Female) | 0.47 | 0.30-0.73 | 0.00 | 0.41 | 0.25-0.67 | 0.00 | 0.44 | 0.23-0.85 | 0.02 | / | / | 0.18 |
| **Size, mm**  (≤10 / >10) | 3.00 | 2.03-4.43 | 0.00 | 3.12 | 2.08-4.66 | 0.00 | 9.59 | 4.43-20.76 | 0.00 | 9.38 | 4.28-20.56 | 0.00 |
| **Hashimoto’s thyroiditis**  (Yes / No) | 0.90 | 0.61-1.34 | 0.62 |  |  |  | 0.78 | 0.40-1.50 | 0.45 |  |  |  |
| **Multifocality**  (No / Yes) | 1.65 | 1.09-2.49 | 0.02 | / | / | 0.07 | 2.51 | 1.34-4.71 | 0.00 | 2.42 | 1.19-4.92 | 0.02 |
| **Extrathyroidal extension**  (No / Yes) | 1.57 | 0.26-9.50 | 0.62 |  |  |  | 4.51 | 0.62-32.81 | 0.14 | / | / | 0.47 |

OR, odds ratio; CI, confidence interval
